# Evaluating the time trends in prevalence of exomphalos in 14 cities of Liaoning province, 2006 to 2015

**DOI:** 10.1038/srep32901

**Published:** 2016-09-08

**Authors:** Ting-Ting Gong, Qi-Jun Wu, Yan-Ling Chen, Cheng-Zhi Jiang, Jing Li, Li-Li Li, Cai-Xia Liu, Da Li, Chen Zhou, Yan-Hong Huang

**Affiliations:** 1 Department of Obstetrics and Gynecology, Shengjing Hospital of China Medical University, Shenyang, China; 2 Department of Clinical Epidemiology, Shengjing Hospital of China Medical University, Shenyang, China; 3 Liaoning Women and Children’s Health Hospital, Shenyang, China; 4 School of Environmental and Chemical Engineering, Shenyang Ligong University, Shenyang, China; 5 Department of science and education, Shenyang Women and Children Health Care Centre, Shenyang, China; 6 Department of children’s health prevention, Shenyang Women and Children Health Care Centre, Shenyang, China; 7 Department of Information Statistics, Shenyang Women and Children Health Care Centre, Shenyang, China

## Abstract

To evaluate time trends of exomphalos prevalence using a large population-based study with cases identified by the Liaoning Birth Defects Registry including 14 cities over the course of a 10-year period. Exomphalos prevalence, percent change, annual percent change (APC), and contribution rates of each city were calculated. Additionally, epidemiological characteristics of this malformation were described. We observed 516 cases of exomphalos among 3,248,954 live births. Birth prevalence of exomphalos was 1.59 per 10,000 live births with non-significant change during the observational period (APC = −1.19%, *P* = 0.48). However, significantly decreasing trends were noticed in three cities: Fushun (APC = −9.15%, *P* = 0.03), Benxi (APC = −11.49%, *P* = 0.05), and Yingkou (APC = −16.47%, *P* = 0.04), contributing 62.77% of the decreasing trend of overall prevalence. The mean maternal age, gestational age, and birth weight was 28.4 years (standard deviation [SD], 6.1 years), 25.6 weeks (SD, 8.6 weeks), and 1236.2 gram (SD, 1164.4 gram). For time of diagnosis, 79.8% (n = 412) cases were diagnosed during pregnancy. In summary, the prevalence of exomphalos in Liaoning province did not change remarkably during 2006 to 2015. Future studies are warranted to investigate the risk factors and create prevention strategies for this disease.

Exomphalos (also known as omphalocele) is a defect in the development of the muscles of the abdominal wall, which results in the intestines, and in giant omphalocele the liver or spleen, remaining outside of the abdomen enclosed within a sac^1^. Exomphalos is associated with a substantial risk of infant morbidity and mortality^2^. Although early surgical repair can improve the prognosis of this disease, the economic burden in the first year of life can be considerable^3^. The Centers for Disease Control and Prevention in the United States reported that the mean length of hospital stay for newborns undergoing surgical repair of exomphalos was 32.5 days with an estimated mean hospital charge of $141,724 for services rendered^3^,^4^.

The birth prevalence of exomphalos has been reported as being between 0.9 and 3.8 per 10,000 births in various countries. For example, researchers from the USA estimated that the prevalence was 1.9 to 2.8 per 10,000 live births^3^,^5^,^6^,^7^,^8^,^9^. In contrast, the studies in Europe demonstrated that the prevalence was 0.9 to 3.8 per 10,000 live births^1^,^10^,^11^,^12^,^13^,^14^. Additionally, Tan *et al.*^15^ and Byron-Scott *et al.*^16^ suggested that the prevalence was 2.17 per 10,000 in Singapore and 2.9 per 10,000 births in Australia, respectively. Although the discrepancy of prevalence was observed among these countries, most of these studies found no statistically significant change over their respective time periods^1^,^3^,^5^,^7^,^8^,^10^,^11^,^12^,^13^,^14^,^15^,^16^.

Compared to these countries, the studies describing the time trend and prevalence of exomphalos have been limited in China. Zhou *et al.*^17^ utilized the national monitoring database which consisted of 460 hospitals at county level or above county level to report epidemiological data on exomphalos from 1996 to 2000. Additionally, a recent report from Li *et al.*^18^ observed the prevalence of exomphalos was 1.50 per 10,000 live births and no long-term trends found for occurrence of omphalocele in China between 1996 and 2010. While, these aforementioned databases were almost years ago. Notably, to the best of knowledge, no study has demonstrated the time trends and prevalence of exomphalos in China by the data of the past decade. Whether prevalence of this disease has continued to be constant has been still unknown. Notably, there have been no study formally assess the time trend of exomphalos prevalence in Liaoning province which encompasses an area of 145,900 square kilometers and has a population of almost 42 million. Therefore, to address these aforementioned research questions, we examine exomphalos prevalence among infants as well as describe the epidemiological characteristics of this malformation in Liaoning province for the 10-year period from 2006–2015.

## Results

Table 1 presents the number of live births of each city in Liaoning province during the 10-year observational period. During this period, the overall number of live births was highest in 2014 (364,400) but lowest in 2015 (298,437). Additionally, when compared with cities, Shenyang, the capital city of this province, had the largest number of live births in each year. In contrast, Benxi had the smallest number of live births.

The prevalence of exomphalos in each city in Liaoning province was demonstrated in Table 2. During 2006–2015, 516 exomphalos cases were detected among 3,248,954 live births (prevalence rate = 1.59 per 10,000 live births). Shenyang (2.49 per 10,000 live births), Dandong (2.18 per 10,000 live births), and Fushun (2.16 per 10,000 live births) were the top three leading cities in Liaoning province. In contrast, Panjing (0.64 per 10,000 live births), Anshan (0.71 per 10,000 live births), and Yingkou (0.76 per 10,000 live births) were the three cities with lowest exomphalos prevalence.

**Figure 1:**
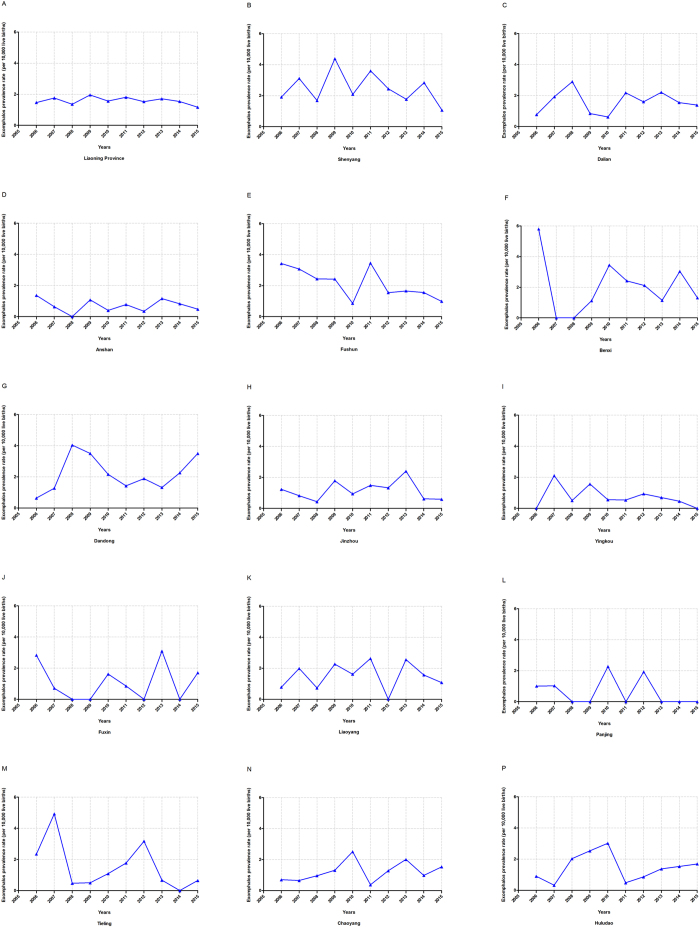
Trends in exomphalos prevalence (per 10,000 live births) of each city in Liaoning province, 2006–2015. (**A**) Liaoning province; (**B**) Shenyang; (**C**) Dalian; (**D**) Anshan; (**E**) Fushun; (**F**) Benxi; (**G**) Dandong; (**H**) Jinzhou; (**I**) Yingkou; (**J**) Fuxin; (**K**) Liaoyang; (**K**) Panjing; (**L**) Tieling; (**M**) Chaoyang; (**N**) Huludao.

Figure 1 depicts the time trend of exomphalos prevalence in each city of Liaoning Province from 2006 to 2015. Although the overall prevalence decreased from 1.47 to 1.17 per 10,000 live births, the result of APC model did not show statistical significance (APC = 1.19; 95% CI: −4.99 to 2.76) (Table 3). However, among these 14 cities, we observed significant decreasing trends in three cities, Fushun (APC = −9.15%; 95% CI: −16.78 to −0.83), Benxi (APC = −11.49%; 95% CI: −21.12 to −0.67), and Yingkou (APC = −16.47%; 95% CI: −28.92 to −1.84). Notably, these aforementioned cities were the three major ones which contributed almost 62.77% of the decreasing trend of exomphalos prevalence in Liaoning province (Table 4).

Detailed epidemiological characteristics of mothers and infants with exomphalos are presented in Table 5. The majority of these cases (79.8%) were diagnosed during the pregnancy. Maternal age over 30 years accounted for 38.8% of the all exomphalos cases. Han was the major maternal race of these mother (89.0%). Among these infants, the mean gestational ages and birth weight were 25.6 weeks (standard deviation [SD], 8.6 weeks) and 1236.2 g (SD, 1164.4 g), respectively. In these exomphalos infants, 49.2% of the infants were male and 42.8% were female.

## Discussion

To our knowledge, this report is one of the few reports from China, the biggest developing countries, evaluating the time trend of exomphalos prevalence as well as identifying the epidemiological characteristics. Although non-significant long-term trends were observed for the 10-year period from 2006 through 2015 in Liaoning province, we observed significant decreasing trends in three cities (Fushun, Benxi, and Yingkou). Additionally, analyses of epidemiological characteristics of all exomphalos cases presented that 79.8% cases were diagnosed during pregnancy and only 20.7% cases were given live birth. Notably, maternal age over 25 years accounted for 74.6% of the all exomphalos cases.

The overall prevalence of exomphalos for Liaoning Province in our study from 2006 to 2015 was 1.59 cases per 10,000 live births. This result was similar to two previous studies in China (1.50 per 10,000 live births between 1996 and 2010 and 1.52 per 10,000 live births between 1996 and 2000)^17^,^18^. Similar prevalence were also observed in several countries. For example, on the basis of 12 state population-based birth defects registries in the United States, Marshall *et al.*^3^ reported that the overall prevalence of exomphalos was 1.92 per 10,000 live births during 1995 to 2005 (decreasing from 1.90 to 1.71 per 10,000 births in 2005). In addition, Stoll *et al.*^19^ found the overall exomphalos prevalence was 2.18 per 10,000 live births during 1979 to 1988 in France. However, on the basis of data from the British Isles Network of Congenital Anomaly Registers from 2005 to 2011, the exomphalos prevalence was 3.8 per 10,000 live births which was relatively higher than that of our study. Notably, Springett *et al.*^1^ observed significantly higher birth prevalence of exomphalos than their previous report using the data from 1987 to 1995^20^. Furthermore, an increasing trend was observed in Austria (from 1.10 cases per 10,000 births in 1974–1979 to 4.20 cases per 10,000 births in 2005–2009) and North America (from 1.88 cases per 10,000 births in 1980–1984 to 4.00 cases per 10,000 births in 2005–2009).

Although non-significant trend was observed in Liaoning province during 2006 to 2015, significant results were observed in three cities, Fushun (APC = −9.15%, P = 0.03), Benxi (APC = −11.49%, P = 0.05), and Yingkou (APC = −16.47%, P = 0.04), which contributed 62.77% of the decreasing trend of overall exomphalos prevalence (Table 4). Additionally, we observed significant geographical variation in prevalence within Liaoning. For example, Shenyang had four-fold prevalence than that of Panjing. This difference could not be attributed to the ascertainment of exomphalos since all the cases were reviewed and confirmed through a group of state-level experts in medical genetics and pediatrics. Previous findings demonstrated a higher risk of omphalocele in residents of rural New York than those living in urban areas of the state^6^, and another study showed large geographic variations in Europe^10^. Furthermore, development of a region may be associated with many environmental exposures including maternal age at delivery^21^,^22^,^23^, socio-economic status^23^,^24^, maternal diet and drug use during pregnancy^23^,^25^,^26^ which were potential risk factors for exomphalos. Therefore, different development of these cities could possibly explain the differences. However, since the access on the data, we could not investigate these hypotheses. Future studies are warranted to provide more evidence of development of area (income level) and further focus on these issues.

The strength of this report include the use of population-based data collected through the province. This was a relatively large time period of data (10 years) as well as accurate results that provided a more recent report on the status of exomphalos prevalence in 14 cities of Liaoning province, with data up to 2015. Compared with previous reports, our study described the prevalence and time trend of exomphalos in all 14 cities instead of pooling them which provided the possibility of comparison between cities. Notably, this report described the time trend of exomphalos prevalence on the basis of the data from one of the most important Provinces in China, which provided the valuable evidence from developing countries. Our findings need to be interpreted with caution in the context of their limitations. First, we had no access to the demographic factors for all live births in Liaoning Province (e.g., maternal age, race/ethnicity), which hindered our ability to investigate the potential causes of the trends. Although we described the epidemiological characteristics for exomphalos infants and their mother from 2006 to 2015 (Table 5), we could hardly confirm the phenomenon that the prevalence of exomphalos was especially higher in specific subgroups (e.g., younger mothers). Additionally, we could not get the prevalence of Liaoning province as far back as 2006 since the access to the data. However, our report provided the trend of exomphalos prevalence in Liaoning province on the basis of the recent decade which has been very limited in developing countries. Second, the maximum time to diagnosis for exomphalos was the seventh day after birth^27^. We did not include exomphalos confirmed after the seventh day, which might have led to slightly lower prevalence in our study than in studies that include longer periods for confirmed diagnoses.

In conclusion, a non-significant trend of exomphalos prevalence was found in Liaoning Province over the past decade which was important for policy makers to understand the recent dynamics of exomphalos prevalence. Future studies, especially cooperating with the public health departments, should identify and focus on lifestyle factors and explore their relationship with exomphalos prevalence over time. Monitoring the populations exposed to these risk factors and taking preventive interventions should be a high national priority in public health in China.

## Material and Methods

### Study population and data source

Liaoning Women and Children’s Health Hospital is one of the sole obstetrical and gynecological hospitals for the province of Liaoning. It has also been a comprehensive care institution, responsible for women’s and children’s health care guidance. Data from 2006 to 2015 were retrieved from the maternal and child health certificate registry of Liaoning Province, which was maintained by this hospital. Hospital-delivered live-born and stillborn infants are included in this registry as the monitored subjects. This registry covers all 14 cities of the province (Shenyang, Dalian, Anshan, Fushun, Benxi, Dandong, Jinzhou, Yingkou, Fuxin, Liaoyang, Panjing, Tieling, Chaoyang, Huludao), with approximately 42 million inhabitants. Liaoning Province is one of the 31 provinces providing data to the national birth defects surveillance database maintained by the Chinese Birth Defects Monitoring Network. All congenital malformation data are regularly uploaded to the online reporting system for maternal and child health surveillance by specialized staff in Liaoning Women and Children’s Health Hospital. The maximum time to provide a diagnosis of a congenital malformation is the seventh day after birth^27^.

The details procedures of data collection were described in previous report^27^. Briefly, a ‘Birth Defects Register Form’ was used to collect the related information on the infants with exomphalos. A ‘Birth Defects Register Form’ is used for collecting the information including demographic characteristics, clinical features, and obstetric items. Each neonate (or terminated fetus) was examined immediately after birth by trained health-care professionals, to screen for congenital malformations. For suspected cases that were diagnosed through prenatal ultrasound scans, case ascertainment after termination or examination after the birth was requested. Once an exomphalos case was identified and confirmed at the monitored hospital by experts in the departments of pediatrics or obstetrics or ultrasound, the mother of the infant was interviewed by the staff in order to complete the aforementioned register form. Subsequently, the ‘Birth Defects Register Form’ was first submitted to the local maternal and child health facility and then to the provincial maternal and child health hospital, which is Liaoning Women and Children’s Health Hospital. The data of these cases were reviewed and confirmed by a group of state-level experts in medical genetics and pediatrics^27^.

Exomphalos was defined as a midline abdominal wall defect, which was limited to an open umbilical cord, according to the World Health Organization’s International Classification of Diseases, 10th Revision^2^. All isolated, multiple cases of omphalocele were included in our analysis. The birth prevalence of omphalocele was expressed per 10,000 live births. The denominator was based exclusively on the total number of live births and data were obtained primarily from the Liaoning Women and Children’s Health Hospital. For suspected exomphalos cases that were diagnosed through prenatal ultrasound scans, case ascertainment after termination or examination after the birth were requested. Therefore, five hundred and sixteen cases were identified. Additionally, the total number of live births in the study window was 3,248,954.

The data quality control was described in detail in previous literature^27^. In brief, according to the program manual to ensure high quality data, the disease diagnosis, data collection, data checking, and medical records were verified by the expert group at each level. In addition, an independent retrospective survey was organized by the experts to find deficiencies and inaccuracies in the data^27^. This study was conducted in compliance with local and national regulations and it was approved by the Institutional Review Board of Liaoning Women and Children’s Health Hospital.

### Statistical analysis

Exomphalos prevalence were calculated for nine 1-year time intervals from 2006 to 2015. The annual percentage change for exomphalos prevalence was used to quantify the time trends^28^,^29^,^30^,^31^. In order to look specifically at time trends, A regression line was fitted to the natural logarithm of the rates, weighted by the number of cases, i.e. y = α + βx + ε, where y = ln (rate) and x = calendar year, and then the APC was calculated as 100×(e^*β*^−1). The 95% confidence interval (CI) of the annual percentage change was calculated by the methods for population-based cancer statistics recommended by the National Cancer Institute (http://seer.cancer.gov/seerstat/WebHelp/seerstat.htm#Trend_Algorithms.htm)^32^. We also calculated the relative contributions for rate changes which provide us for determining the contributions from individual city made to the overall trend^29^,^30^,^32^. Additionally, we described the individual charts of index cases for clinical course (time of diagnosis) and more detailed demographic characteristics of mother (maternal age, number of pregnancy and birth, race, income level, and education level) and infant (gestational age, birth weight, sex, and multiple birth). All analyses were conducted using SPSS for Windows (version 22, SPSS Inc, Chicago, IL, USA). All statistical tests were two-sided, and *P*-values less than 0.05 were considered statistically significant.

## Additional Information

**How to cite this article**: Gong, T.-T. *et al.* Evaluating the time trends in prevalence of exomphalos in 14 cities of Liaoning province, 2006 to 2015. *Sci. Rep.* **6**, 32901; doi: 10.1038/srep32901 (2016).

## Figures and Tables

**Table 1:** The number of live births in each city in Liaoning province, 2006 to 2015.

City	Year										Overall
	2006	2007	2008	2009	2010	2011	2012	2013	2014	2015	
Liaoning Province	306,734	341,432	330,414	321,353	307,826	304,079	353,108	321,171	364,400	298,437	3,248,954
Shenyang	52,256	61,108	59,196	59,200	57,521	58,335	69,721	67,854	80,997	65,118	631,306
Dalian	38,744	46,652	48,309	47,900	48,774	50,490	62,324	58,722	71,178	57,641	530,734
Anshan	29,270	31,305	29,647	27,721	25,184	25,603	28,790	25,855	36,171	20,798	280,344
Fushun	11,661	12,997	12,314	12,337	11,638	11,556	12,942	12,016	12,845	10,138	120,444
Benxi	8,620	9,435	8,759	8,842	8,696	8,261	9,440	8,700	9,857	7,627	88,237
Dandong	15,710	15,725	14,836	14,274	13,894	14,038	15,895	15,111	17,718	14,278	151,479
Jinzhou	24,293	24,261	23,149	22,342	21,255	20,098	22,559	20,860	16,137	16,985	211,939
Yingkou	16,987	18,924	19,667	19,070	17,947	18,484	21,309	14,224	21,684	16,515	184,811
Fuxin	14,158	14,142	13,353	13,322	12,370	11,800	13,050	9,662	9,121	11,752	122,730
Liaoyang	12,888	15,039	13,754	13,200	12,331	11,386	13,296	11,702	12,747	9,251	125,594
Panjing	9,887	9,669	10,134	9,009	8,800	8,867	10,362	9,644	8,276	9,197	93,845
Tieling	21,263	20,298	21,456	19,854	18,421	16,945	18,938	14,960	17,389	15,269	184,793
Chaoyang	28,669	30,980	31,168	30,574	27,837	27,207	31236	29,919	30,646	26,083	294,319
Huludao	22,328	30,897	24,672	23,708	23,158	21,009	23246	21,942	19,634	17,785	228,379

**Table 2:** The prevalence of exomphalos in each city in Liaoning province, 2006 to 2015 (per 10,000 births).

City	Year										Overall
	2006	2007	2008	2009	2010	2011	2012	2013	2014	2015	
Liaoning Province	1.47	1.76	1.36	1.96	1.56	1.81	1.53	1.71	1.54	1.17	1.59
Shenyang	1.91	3.11	1.69	4.39	2.09	3.60	2.44	1.77	2.84	1.07	2.49
Dalian	0.77	1.93	2.90	0.84	0.62	2.18	1.60	2.21	1.55	1.39	1.62
Anshan	1.37	0.64	0.00	1.08	0.40	0.78	0.35	1.16	0.83	0.48	0.71
Fushun	3.43	3.08	2.44	2.43	0.86	3.46	1.55	1.66	1.56	0.99	2.16
Benxi	5.80	0.00	0.00	1.13	3.45	2.42	2.12	1.15	3.04	1.31	2.04
Dandong	0.64	1.27	4.04	3.50	2.16	1.42	1.89	1.32	2.26	3.50	2.18
Jinzhou	1.23	0.82	0.43	1.79	0.94	1.49	1.33	2.40	0.62	0.59	1.18
Yingkou	0.00	2.11	0.51	1.57	0.56	0.54	0.94	0.70	0.46	0.00	0.76
Fuxin	2.83	0.71	0.00	0.00	1.62	0.85	0.00	3.10	0.00	1.70	1.06
Liaoyang	0.78	1.99	0.73	2.27	1.62	2.63	0.00	2.56	1.57	1.08	1.51
Panjing	1.01	1.03	0.00	0.00	2.27	0.00	1.93	0.00	0.00	0.00	0.64
Tieling	2.35	4.93	0.47	0.50	1.09	1.77	3.17	0.67	0.00	0.65	1.62
Chaoyang	0.70	0.65	0.96	1.31	2.51	0.37	1.28	2.01	0.98	1.53	1.22
Huludao	0.90	0.32	2.03	2.53	3.02	0.48	0.86	1.37	1.53	1.69	1.44

**Table 3:** Trends in exomphalos prevalence in each city of Liaoning during 2006–2015.

Exomphalos	2006		2015		PC† (%)	APC† (%)	*P* value	95% CI
	Case	Rate*	Case	Rate*				
Overall	45	1.47	35	1.17	−20.06	−1.19	0.48	−4.99, 2.76
Shenyang	10	1.91	7	1.07	−43.83	−3.34	0.50	−13.27, 7.72
Dalian	3	0.77	8	1.39	79.24	−1.49	0.77	−12.22, 10.55
Anshan	4	1.37	1	0.48	−64.82	−4.59	0.34	−14.19, 6.09
Fushun	4	3.43	1	0.99	−71.24	−9.15	0.03	−16.78, −0.83
Benxi	5	5.80	1	1.31	−77.40	−11.49	0.05	−21.12, −0.67
Dandong	1	0.64	5	3.50	450.15	−1.00	0.87	−13.39, 13.17
Jinzhou	3	1.23	1	0.59	−52.32	4.08	0.53	−9.58, 19.80
Yingkou	0	0.00	0	0.00	N/A	−16.47	0.04	−28.92, −1.84
Fuxin	4	2.83	2	1.70	−39.76	−0.70	0.93	−15.69, 16.97
Liaoyang	1	0.78	1	1.08	39.31	3.05	0.59	−9.02, 16.71
Panjing	1	1.01	0	0.00	−100.00	13.54	0.16	−0.44, 29.49
Tieling	5	2.35	1	0.65	−72.15	−10.86	0.26	−28.23, 10.71
Chaoyang	2	0.70	4	1.53	119.83	6.29	0.34	−7.23, 21.78
Huludao	2	0.90	3	1.69	88.32	−0.80	0.91	−16.36, 17.66

APC, annual percent change; CI, confidence interval; N/A, not available; PC, percent change.

^*^Gastroschisis prevalence were expressed as per 10,000 live births.

^†^Percent change and annual percent change between 2006 and 2015 was calculated by the gastroschisis prevalence.

**Table 4:** The relative contributions of decreasing trend of exomphalos prevalence of each city in Liaoning Province during 2006–2015.

City	Decreasing trend		Increasing trend	
	β	Contribution rate (%)	β	Contribution rate (%)
Shenyang	−0.03	5.36	—	—
Dalian	−0.02	2.37	—	—
Anshan	−0.05	7.41	—	—
Fushun	−0.10	15.14	—	—
Benxi	−0.12	19.24	—	—
Dandong	−0.01	1.58	—	—
Jinzhou	—	—	0.04	15.50
Yingkou	−0.18	28.39	—	—
Fuxin	−0.01	1.10	—	—
Liaoyang	—	—	0.03	11.63
Panjing	—	—	0.13	49.22
Tieling	−0.12	18.14	—	—
Chaoyang	—	—	0.06	23.64
Huludao	−0.01	1.26	—	—

**Table 5:** Demographic characteristics for exomphalos infants and their mother during 2006–2015.

Characteristics	Exomphalos
No. of cases	516
Time of diagnosis (%)	
During pregnancy	412 (79.8)
Within 7 days after delivery	104 (20.2)
Mother	
Maternal age (%)	
<25	131 (25.4)
25–30	185 (35.8)
≥30	200 (38.8)
No. of pregnancy (SD)	1.8 (1.2)
No. of live births (SD)	0.7 (0.7)
Race (%)	
Han	459 (89.0)
Others	57 (11.0)
Income level† (%)	
< 600 yuan	28 (5.4)
600–1200 yuan	69 (13.4)
1200–2400 yuan	110 (21.3)
≥ 2400 yuan	309 (59.9)
Education level (%)	
Elementary school or less	30 (5.8)
Middle school	239 (46.3)
High school	114 (22.1)
College or above	133 (25.8)
Infant	
Gestational age, week (SD)	25.6 (8.6)
Birth weight, gram (SD)	1236.2 (1164.4)
Sex (%)	
Male	254 (49.2)
Female	221 (42.8)
Unknown	41 (8.0)
Multiple births (%)	
Yes	20 (3.9)
No	496 (96.1)

SD, standard deviation.

^†^Income level was presented as per person per year.
